# *Salmonella enterica* Serovar Abony Outbreak Caused by Clone of Reference Strain WDCM 00029, Chile, 2024

**DOI:** 10.3201/eid3101.241012

**Published:** 2025-01

**Authors:** Alejandro Piña-Iturbe, Diego Fredes-García, Patricia García, Lorena Porte, Timothy J. Johnson, Randall S. Singer, Magaly Toro, José M. Munita, Andrea I. Moreno-Switt

**Affiliations:** Pontificia Universidad Católica de Chile, Santiago, Chile (A. Piña-Iturbe, D. Fredes-García, P. García, A.I. Moreno-Switt); Clínica Alemana de Santiago, Santiago, Chile (L. Porte); Universidad del Desarrollo, Santiago, Chile (L. Porte, J.M. Munita); University of Minnesota, Saint Paul, Minnesota, USA (T.J. Johnson, R.S. Singer); University of Maryland, College Park, Maryland, USA (M. Toro)

**Keywords:** *Salmonella* *enterica*, serovar Abony, WDCM 00029, bacteria, enteric infections, food safety, outbreak, Chile

## Abstract

A *Salmonella enterica* serovar Abony outbreak occurred during January–April 2024 in Chile. Genomic evidence indicated that the outbreak strain was a clone of reference strain WDCM 00029, which is routinely used in microbiological quality control tests. When rare or unreported serovars cause human infections, clinicians and health authorities should request strain characterization.

Nontyphoidal *Salmonella enterica* is a major cause of foodborne disease worldwide (*1*). Although a few serovars, such as Enteritidis and Typhimurium, produce most infections (*2*,*3*), uncommon serovars can cause clinical cases. Characterization may contribute to early identification of emerging strains. We report a multiregional outbreak of salmonellosis caused by *Salmonella* Abony and characterization of clinical isolates collected during the outbreak. 

During January 19–March 16, 2024, two healthcare centers in Santiago, Chile, diagnosed 134 human salmonellosis cases: 29 at UC-Christus and 105 at Clínica Alemana. All isolates were submitted to Instituto de Salud Pública de Chile for serotyping; serovar Abony (antigenic formula 1,4,[5],12:b:e,n,x) was found in 57% (56/97) of cases with culture (Appendix 1 Figure 1). Among those cases, 33 (58.9%) were in male patients and 23 (41.1%) in female patients; 40 (71.4%) patients were <18 years of age, 17 (30.4%) required hospitalization, and 10 (17.9%) had bacteremia (Appendix 2 Table 1).

Whole-genome sequencing was performed for 18 of 56 outbreak isolates, 13 from UC-Christus and 5 from Clínica Alemana (Appendix 1 Figure 1). Isolates comprised 6 blood, 2 urine, and 10 feces samples. Hierarchical clustering (HC) of global *Salmonella* Abony genomes identified 150 HC50 (<50 core genome multilocus sequence typing allele differences) clusters; outbreak isolates belonged to the HC50_20673 cluster (Appendix 1 Figure 2; Appendix 2 Table 2). That cluster encompassed isolates from the United Kingdom, United States, Brazil, Nigeria, and France collected during 2008–2024. A core single-nucleotide polymorphism (SNP) phylogeny grouped all HC50_20673 genomes into 3 clades corresponding to 3 HC10 clusters (Figure 1). The genomes from Chile grouped within HC10_20673, differing by only 0–3 pairwise SNP differences. That cluster also included 4 genomes from reference strain *Salmonella* Abony WDCM 00029 (*4*) (Figure 1; Appendix 2 Table 2). The 4 WDCM 00029 genomes differed by 0–19 SNPs from the remaining HC10_20673 isolates and by 0–7 SNPs from the isolates from Chile, indicating high relatedness (Appendix 2 Table 3).

**Figure 1:**
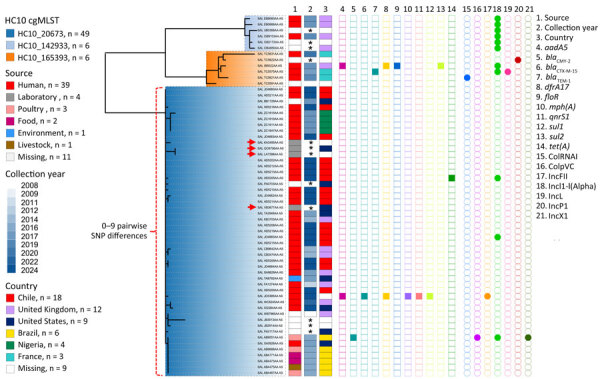
Phylogenetic analysis of *Salmonella enterica* serovar Abony from an outbreak caused by a WDCM 00029 Clone, Chile, 2024. A core SNP maximum-likelihood phylogenetic tree was constructed by using RAxML version 8 (https://github.com/stamatak/standard-RAxML) and genomes of *Salmonella* Abony isolates from the HC50_20673 cluster with the ATCC 6017 genome as the reference (Enterobase Barcode SAL_BA5138AA; Sequence Read Archive accession no. SRR1786283). The tree was constructed by using the Enterobase pipelines refMasker, refMapper, refMapperMatrix, and matrix_phylogeny, which together masked repeated regions, tandem repeats, and CRISPR regions in the reference genome, aligned genomes to reference, called nonrepetitive core SNPs, and built the maximum likelihood tree. Metadata regarding HC10 clusters (<10 allele differences) include isolation source, collection year, country of origin, antibiotic drug resistance genes (AMRFinderPlus version 3.12.8; database version 2024-05-02.2; https://www.ncbi.nlm.nih.gov/pathogens/antimicrobial-resistance/AMRFinder), and plasmid replicons (ABRicate version 1.0.1, https://github.com/tseemann/abricate; PlasmidFinder database, updated June 4, 2024). Red arrows indicate the WDCM 00029 genomes found in the public databases (Enterobase, https://enterobase.warwick.ac.uk/species/index/senterica; National Center for Biotechnology Information Sequence Read Archive, https://www.ncbi.nlm.nih.gov/sra) that were made public in 2013 (accession no. SRR955283), 2016 (accession no. SRR1815498), 2019 (accession no. SRR8599079), and 2021 (accession no. SRR15145673). Asterisks (*) in the collection year column indicate that information was missing. The figure was made by using iTOL version 6.9 (https://itol.embl.de). ATCC, American Type Culture Collection; cgMLST, core genome multilocus sequence typing; CRISPR, clustered regularly interspaced short palindromic repeats; SNP, single-nucleotide polymorphism.

To confirm the high genomic similarity was not limited to the core genome, we calculated average nucleotide identity and alignment fraction for all HC50_20673 isolates using the WDCM 00029 genome provided by the American Type Culture Collection (strain BAA-2162; https://www.atcc.org) (*5*) as the reference (Figure 2, panel A). Of note, isolates from Chile had median alignment fraction (97.85%) and average nucleotide identity (99.99%) values greater than those of the other HC10 clusters (p<0.0001) (Figure 2, panels B, C), in line with core SNP data and further suggesting an almost complete genomic identity between WDCM 00029 and the outbreak genomes. We observed similar findings when we included all HC10_20673 genomes, except WDCM 00029 genomes, in the analysis (Appendix 1 Figure 3).

**Figure 2:**
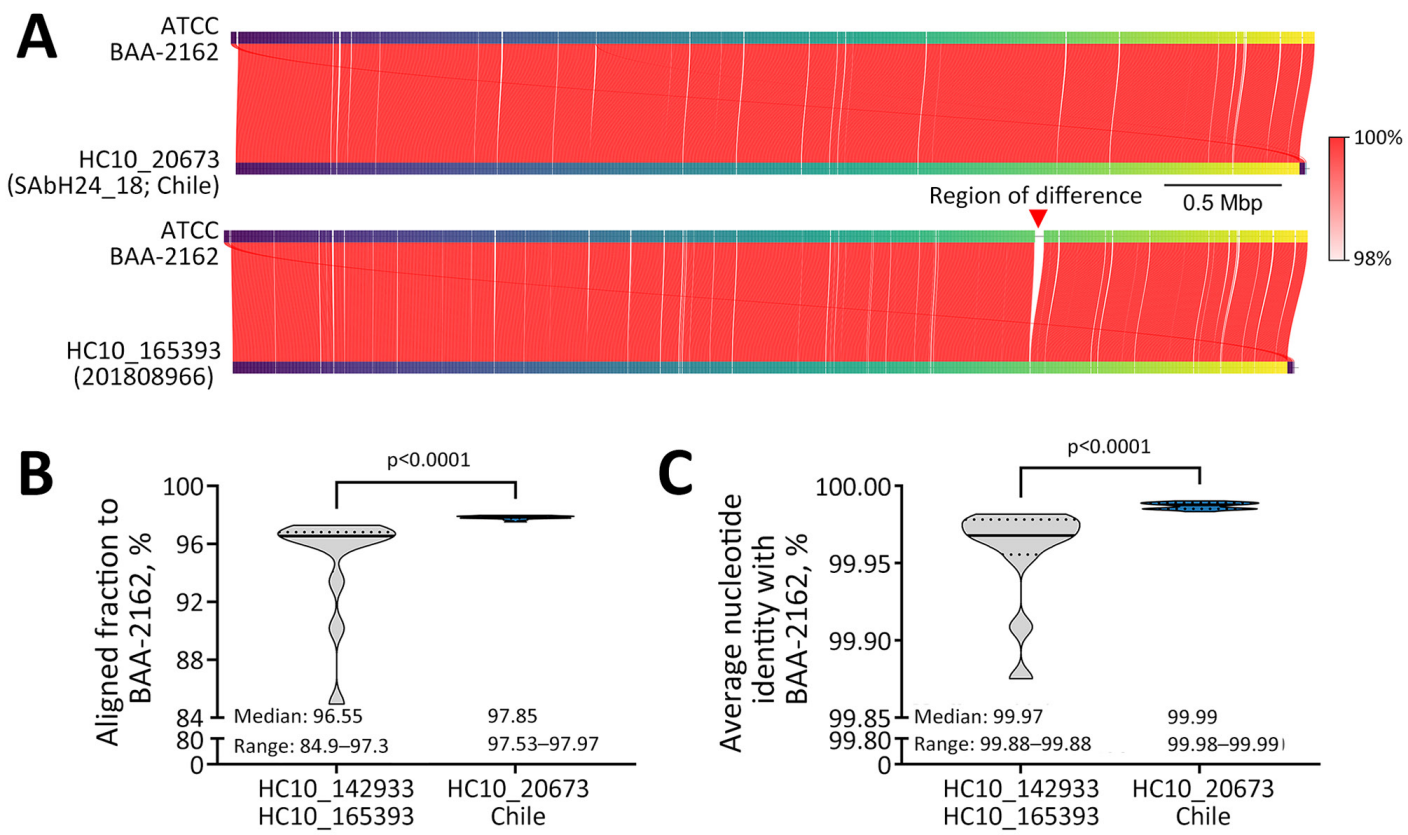
Whole-genome comparisons of *Salmonella enterica* serovar Abony from an outbreak caused by a WDCM 00029 clone, Chile, 2024. A) Example whole-genome comparison between *Salmonella* Abony WDCM 00029 (genome provided by ATCC; strain BAA-2162) and an isolate from the HC10_20673 cluster (strain SAbH24_18) from Chile, or the most closely related isolate (strain 201808966) outside the HC10_20673 cluster (mean difference to HC10_20673 isolates: 73 SNPs). Red lines connect regions of genome identity between each pair of compared genomes, with color indicating the percent identity. The red vertical arrow points to a region of difference between the compared genomes. B, C) Truncated violin plots of AF (B) and ANI (C) to the WDCM 00029 genome of the *Salmonella* Abony isolates (n = 18 genomes) from Chile and other isolates from the same HC50 cluster (HC10_142933 and HC10_165393; n = 12 genomes). In the violin plots, black horizontal lines represent medians and dotted lines represent 25% and 75% quartiles. Differences between the median values were assessed by using Mann-Whitney tests. AF and ANI calculations were made with FastANI version 1.34 (https://github.com/ParBliSS/FastANI). AF, alignment fraction; ANI, average nucleotide identity; ATCC, American Type Culture Collection; SNP, single-nucleotide polymorphism.

According to official data requested from the Chile government (Appendix 1 Figure 4; Appendix 2 Table 4), 287 *Salmonella* Abony isolates were collected during January 24–April 21 from 12 of 16 administrative regions of Chile, corresponding to infections occurring in persons 0–82 years old. Most (79.8%; 229/287) isolates came from Región Metropolitana, and 57.5% (165/287) were obtained during February 2024. Because we did not have additional epidemiologic information (e.g., food consumed), we did not investigate the source of the outbreak.

Previous studies from Brazil and Nigeria also reported human *Salmonella* Abony infections, and cases from Brazil were linked to consumption of food containing chicken meat (*6*,*7*). We found those isolates also belonged to the HC10_20673 cluster and were closely related to WDCM 00029 genomes (1–9 core SNPs differences) (Figure 1; Appendix 2 Table 3). One isolate from Brazil was resistant to third-generation cephalosporins because of a *bla*_CMY-2_ –carrying IncI1 plasmid (Figure 1). Moreover, 2 isolates from Chile and 1 isolate from the National Center for Biotechnology Information Pathogen Detection database (https://www.ncbi.nlm.nih.gov/pathogens; strain PNUSAS428168, SNP cluster PDS000001617.32) also carried IncI1 and IncFII plasmids. Strain PNUSAS428168 harbored the *qnrS1* gene involved in resistance to fluoroquinolones and *bla*_CTX-M-15_ gene involved in resistance to third-generation cephalosporins (Figure 1), highlighting the capacity of HC10_20673 *Salmonella* Abony to acquire plasmids conferring resistance to first-line antibiotic drugs used for treating severe salmonellosis.

*Salmonella* Abony WDCM 00029 (also known as strains BAA-2162, NCTC 6017, CIP 80.39, CECT 545, and DSM 4224, among others) is a strain with >80 years of history. Originally isolated from human feces in Hungary before 1940, it was part of Fritz Kauffmann’s collection and was later deposited in different culture collections (*4*,*5*,*8*). WDCM 00029 is widely used as a control strain for testing culture media performance, detailed in pharmacopeial texts from the United States, Europe, and Japan that are accepted by the International Council of Harmonization (*9*,*10*). Accordingly, WDCM 00029 is sold by many suppliers as certified lyophilized or ready-to-use reference material for quality control of food, water, and environmental testing (Appendix 2 Table 5).

In summary, evidence suggests the 2024 *Salmonella* Abony outbreak in Chile was caused by contamination of an unknown vehicle with the widely used WDCM 00029 reference strain. Our findings raise concerns about safety of bacterial quality control strains. When rare or unreported serovars cause human infections, clinicians and health authorities should request strain characterization.

Appendix 1 Additional information on *Salmonella enterica* serovar Abony outbreak caused by WDCM 00029 clone, Chile, 2024.

Appendix 2 Genomic information used in study of *Salmonella enterica* serovar Abony outbreak caused by WDCM 00029 clone, Chile, 2024.
