# Supplementary material for: Salmonella enterica Serovar Abony Outbreak Caused by Clone of Reference Strain WDCM 00029, Chile, 2024
Source: Emerg Infect Dis. 2025 Jan;31(1):186–9. doi: 10.3201/eid3101.241012 (PMC11682822; doi:10.3201/eid3101.241012)
Supplement: Appendix 1 — Additional information on Salmonella enterica serovar Abony outbreak caused by WDCM 00029 clone, Chile, 2024. [file 24-1012-Techapp-s1.pdf]

Article DOI: https://doi.org/10.3201/eid3101.241012

EID cannot ensure accessibility for supplementary materials supplied by authors. Readers who have difficulty accessing supplementary content should contact the authors for assistance.

Salmonella enterica Serovar Abony Outbreak Caused by a Clone of Reference Strain WDCM 00029, Chile, 2024

Appendix

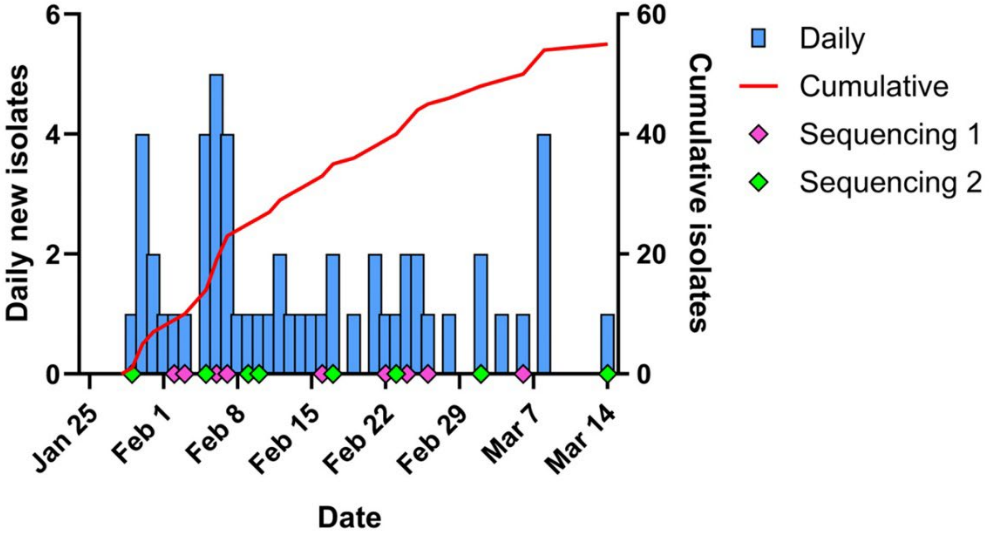

Appendix 1 Figure 1. Temporal distribution of *Salmonella* Abony isolates included in this study. The histogram (blue bars) shows the number of isolates per date of isolation. Information about dates of sample collection date or symptoms onset was not available. Only one isolate per case of infection was included. The red line indicates the cumulative number of *Salmonella* Abony isolates from 0 to 56. The pink and green diamonds indicate the isolation date of the sequenced *Salmonella* Abony (18 isolates) from the 2 microbiology laboratories that were included in this study, respectively.

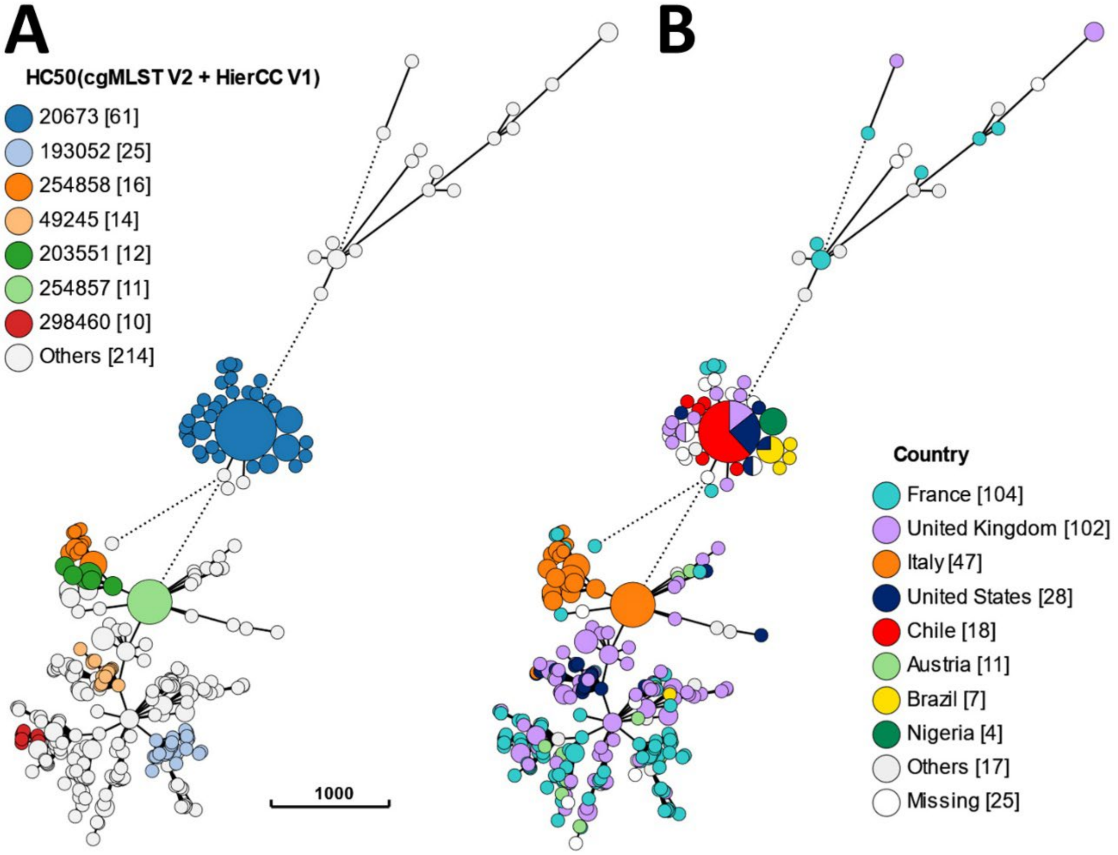

Appendix 1 Figure 2. Structure of *Salmonella* Abony global population. A minimum spanning tree (MST) was constructed in Enterobase based on cgMLST (3002 alleles). Node size is proportional to the number of genomes included. Edges represent the number of allele differences. Nodes separated by more than 1000 allele differences are liked by broken-line edges. Nodes were colored by A) HC50 cluster according to HierCC v1 or B) country of origin. The Chilean *Salmonella* Abony isolates group in the HC50_20673 cluster together with isolates from France, United Kingdom, Brazil, and Nigeria.

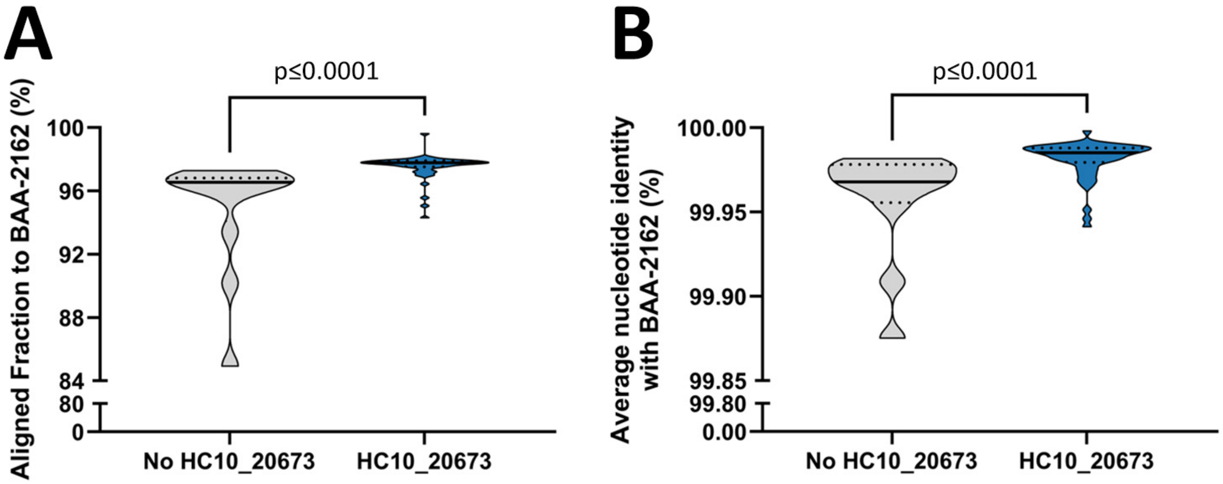

Appendix 1 Figure 3. Aligned fraction and average nucleotide identity of HC50_20673 genomes to the WDCM 00029 genome. Truncated violin plots of A) the aligned genome fraction (AF) and B) average nucleotide identity (ANI) of HC10_20673 and no HC10_20673 (HC10_142933 and HC10_165393) isolates to the WDCM 00029 genome provided by the American Type Culture Collection (ATCC BAA-2162). The black horizontal line and dotted lines in the violin plots represent the median and the 25% and 75% quartiles, respectively. The AF and ANI were calculated with FastANI v1.34. Differences between the median values were assessed with the Mann-Whitney test (****p<0.0001).

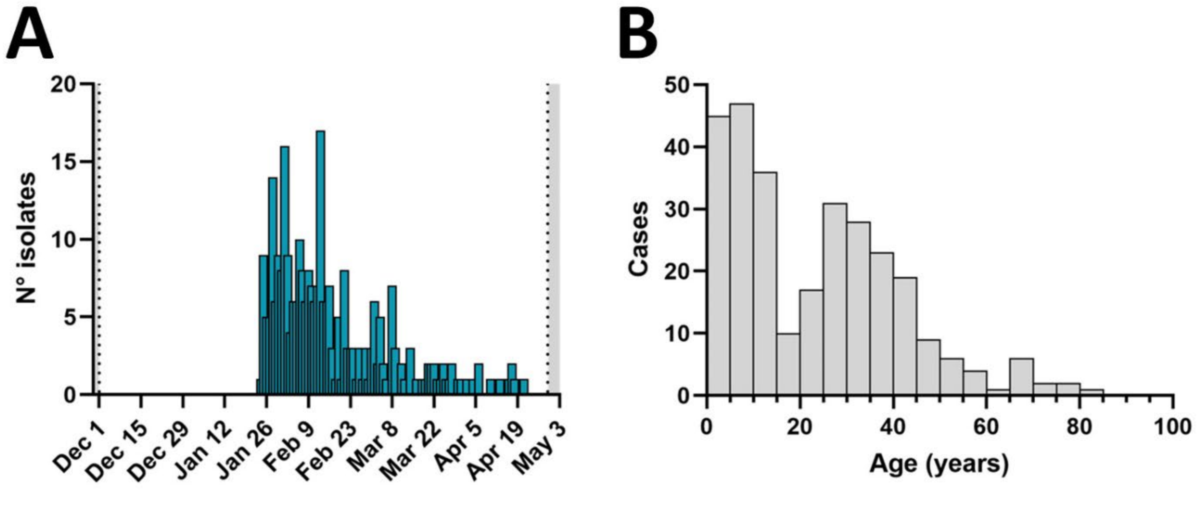

Appendix 1 Figure 4. *Salmonella* Abony isolates received by the Instituto de Salud Pública (ISP) of Chile. A) Histogram of the *Salmonella* Abony isolates received by the ISP (287 isolates) from December 1, 2023 to April 29, 2024, and B) age distribution of infection cases according to data requested to the ISP. Some isolates may be obtained from the same patient.
